# Despite Antagonism *in vitro*, *Pseudomonas aeruginosa* Enhances *Staphylococcus aureus* Colonization in a Murine Lung Infection Model

**DOI:** 10.3389/fmicb.2019.02880

**Published:** 2019-12-13

**Authors:** Guillaume Millette, Jean-Philippe Langlois, Eric Brouillette, Eric H. Frost, André M. Cantin, François Malouin

**Affiliations:** ^1^Centre d’Étude et de Valorisation de la Diversité Microbienne, Département de Biologie, Faculté des Sciences, Université de Sherbrooke, Sherbrooke, QC, Canada; ^2^Département de Microbiologie et d’Infectiologie, Faculté de Médecine et de Sciences de la Santé, Université de Sherbrooke, Sherbrooke, QC, Canada; ^3^Service de Pneumologie, Département de Médecine, Faculté de Médecine et des Sciences de la Santé, Université de Sherbrooke, Sherbrooke, QC, Canada

**Keywords:** microbial interactions, oral microbiology, *Pseudomonas*, *Staphylococcus*, quorum sensing, sociomicrobiology, microbiome, genomics

## Abstract

*Staphylococcus aureus* and *Pseudomonas aeruginosa* are prevalent lung pathogens in cystic fibrosis (CF). Whereas co-infection worsens the clinical outcome, prototypical strains are usually antagonistic *in vitro*. We sought to resolve the discrepancy between these *in vitro* and *in vivo* observations. *In vitro*, growth kinetics for co-cultures of co-isolates from CF patients showed that not all *P. aeruginosa* strains affected *S. aureus* viability. On solid media, *S. aureus* slow-growing colonies were visualized around some *P. aeruginosa* strains whether or not *S. aureus* viability was reduced in liquid co-cultures. The *S. aureus–P. aeruginosa* interactions were then characterized in a mouse lung infection model. Lung homogenates were plated on selective media allowing colony counts of either bacterium. Overall, 35 *P. aeruginosa* and 10 *S. aureus* strains (clinical, reference, and mutant strains), for a total of 200 co-infections, were evaluated. We observed that *S. aureus* colonization of lung tissues was promoted by *P. aeruginosa* and even by strains showing antagonism *in vitro*. Promotion was proportional to the extent of *P. aeruginosa* colonization, but no correlation was found with the degree of myeloperoxidase quantification (as marker of inflammation) or with specific virulence-associated factors using known mutant strains of *S. aureus* and *P. aeruginosa*. On the other hand, *P. aeruginosa* significantly increased the expression of two possible cell receptors for *S. aureus*, *i.e*., ICAM-1 and ITGA-5 (marker for integrin α_5_β_1_) in lung tissue, while mono-infections by *S. aureus* did not. This study provides insights on polymicrobial interactions that may influence the progression of CF-associated pulmonary infections.

## Introduction

Cystic fibrosis (CF) is the most common recessive genetic disorder leading to chronic pulmonary infections, gastrointestinal disorders, diabetes, and other health complications. The most severe complications are associated with recurrent lung infections, which are responsible for high morbidity and mortality (Cystic Fibrosis Canada, 2018). A gene defect in the CF transmembrane conductance regulator (CFTR), which is a membrane protein and chloride channel, causes abnormally thick and viscous mucus production in the lung mucosa (Ratjen, 2009; Kreda et al., 2012). This defect affects muco-ciliary clearance, reduces bacterial killing via an impaired HCO_3_ excretion, and thus supports bacterial growth (Castellani and Assael, 2017). Infections lead to inflammation, and the host response stimulates further mucus production (Gómez and Prince, 2007; Ratjen, 2009). The mucus, rich in nutrients and not being efficiently cleared, promotes colonization of opportunistic pathogens (Palmer et al., 2005; Sriramulu et al., 2005). The establishment of this feedback loop results in frequent exacerbations and increasingly reduced lung functions, which eventually lead to death (Lyczak et al., 2002).

Throughout their lives, CF patients will be infected by many opportunistic environmental bacteria, the two most common being *Staphylococcus aureus* and *Pseudomonas aeruginosa*. The following chronological trend occurs in most patients: *S. aureus* typically colonizes younger patients, then its prevalence declines in adulthood. On the contrary, *P. aeruginosa* infections are infrequent in childhood but become predominant later when CF patients reach adulthood. Despite their seemingly sequential appearance, both pathogens remain highly prevalent through all stages of the lives of CF patients, with, respectively, 59.9 and 40.2% of patients infected by *S. aureus* and *P. aeruginosa* (Cystic Fibrosis Canada, 2018). While *P. aeruginosa* infections undoubtedly cause deterioration in patient health (Sadikot et al., 2005; Harun et al., 2016), the contribution of *S. aureus* infections to morbidity and mortality remains controversial with not all studies agreeing whether they can single-handedly worsen prognosis (Junge et al., 2016; Limoli et al., 2016). However, microbial interactions are possible. Cigana et al. (2018) investigated *S. aureus*–*P. aeruginosa* interactions in a murine chronic lung infection model. Following the natural course of infections in CF, mice were first infected with *S. aureus*, causing abscess-like wounds, then further infected by *P. aeruginosa*. *P. aeruginosa* was able to better chronically infect mice that had been pre-infected with *S. aureus*, reminiscent of that observed in CF-afflicted humans. Furthermore, many reports have associated *S. aureus*–*P. aeruginosa* co-infections with a worse clinical outcome for CF patients such as decreased pulmonary function, more frequent exacerbations, and increased mortality (Hubert et al., 2013; Limoli et al., 2016). Given these insights, it appears critical to further investigate the interactions between these microorganisms, to help prevent and treat deleterious co-infections.

*Staphylococcus aureus* small-colony variants (SCVs) are respiratory-deficient variants differing from their prototypical counterparts by their slow growth, alternative expression of virulence genes, and persistence in chronic infections (Moisan et al., 2006; Mitchell et al., 2013). SCVs are frequently associated with chronic infections, including CF lung infections (Kahl et al., 2016). Their ability to persist is mainly due to increased biofilm production and internalization into host cells, allowing them to evade the action of antibiotics and the immune system (Proctor et al., 2006). The alternative sigma factor B (SigB) is an important regulator of virulence in SCVs, and dominate over the quorum-sensing (QS) Agr system, which is responsible for exotoxins and hydrolytic enzyme expression (Novick and Geisinger, 2008; Mitchell et al., 2013). The presence of SCVs was directly associated with a worse respiratory outcome in children with CF (Wolter et al., 2013). Interestingly, *P. aeruginosa* can induce the SCV phenotype in *S. aureus*. *P. aeruginosa* produces a wide variety of QS molecules to coordinate the expression of its virulence factors, motility and extracellular matrix formation (Williams and Cámara, 2009). Among *P. aeruginosa* QS-controlled virulence factors, many such as the elastases, pyocyanin, pyoverdine, hydrogen cyanide, and alkyl quinolones were shown to negatively affect *S. aureus* growth *in vitro* (Machan et al., 1992; Hoffman et al., 2006; Goerke and Wolz, 2010). More specifically, *P. aeruginosa* 2-heptyl-4-hydroxy quinoline *N*-oxide (HQNO) induces the SCV phenotype by acting as a respiratory chain inhibitor for *S. aureus* (Lightbown and Jackson, 1956). HQNO-sensitized *S. aureus* are known to produce more biofilm, and there is a direct correlation between HQNO levels and biofilm production by *S. aureus* (Mitchell et al., 2010). Interactions between *P. aeruginosa* and *S. aureus* during a co-infection in CF patients are likely to occur and these may modulate virulence in unexpected ways.

On the other hand, we previously demonstrated that *P. aeruginosa* and *S. aureus* strains co-isolated from a same CF patient do not always interact as expected for prototypical strains (i.e., prototypical *P. aeruginosa* inducing biofilm production by *S. aureus in vitro*) (Fugère et al., 2014). For instance, a high HQNO production by some *P. aeruginosa* strains does not proportionally induce biofilm production by the co-isolated *S. aureus* strain. This suggests that co-isolates may adapt to each other in order to persist in the lung. Similarly, Limoli et al. (2017) recently demonstrated that *P. aeruginosa* isolates from long-term coinfected patients did not antagonize *S. aureus in vitro*. While such studies show that *P. aeruginosa* does not always antagonize *S. aureus in vitro*, data from co-infection animal models are needed to better understand clinical observations associated with *P. aeruginosa* and *S. aureus* co-infections.

To our knowledge, the impact of *P. aeruginosa* CF clinical strains on *S. aureus* colonization *in vivo* has never been systematically studied. The objective of the present study was to evaluate the circumstances allowing *S. aureus* to colonize and survive in the lung despite the presence of *P. aeruginosa*. We first used *in vitro* models to characterize clinical strains and the types of interactions between *P. aeruginosa* and *S. aureus* and then compared their ability to co-colonize in a murine lung infection model. Our findings show that *S. aureus* clearly profits from the presence of *P. aeruginosa* in a murine lung infection model, whether or not antagonism is seen *in vitro*.

## Materials and Methods

### Ethics Statement

The animal experiments were carried out according to the guidelines of the Canadian Council on Animal Care and the institutional ethics committee on animal experimentation of the *Faculté des Sciences* of *Université de Sherbrooke*, which specifically approved the protocols used for this study (FM2014-02 and FM2018-01B).

### Bacterial Strains and Growth Conditions

*Pseudomonas aeruginosa* PA14 (Rahme et al., 1995) and *S. aureus* CF07-L (Moisan et al., 2006) were used as prototypical control strains. An additional 29 *P. aeruginosa* and 5 *S. aureus* clinical isolates were also used in this study. These isolates were previously characterized and obtained from 32 adult CF patients (Fugère et al., 2014). These included 16 *P. aeruginosa* that were co-isolated with *S. aureus* and 13 that were not (see additional information in Supplementary Table S1). Among these clinical strains, *S. aureus* strains CF6B-L, CF22A-L, CF39A-L, CF54A-L, and CF112A-L were more specifically selected and studied because their biofilm production was not stimulated by their co-isolated *P. aeruginosa* PAC6B, PAC22A, PAC39A, PAC54A, and PAC112A, respectively (Fugère et al., 2014).

To determine the impact of different bacterial components on the *in vivo* colonization of *S. aureus* in the presence of *P. aeruginosa*, different mutant strains from both species were also used in this study. Table 1 shows the relevant characteristics of those mutants and their origin. The *S. aureus* NRS strains were obtained from the Network on Antimicrobial Resistance in *S. aureus* (NARSA). TSA and TSB (BD, Mississauga, ON, Canada) were generally used as growth media. Cation-adjusted Mueller–Hinton broth (CAMHB; BD, Mississauga, ON, Canada) was used in growth kinetics experiments.

**TABLE 1:** *P. aeruginosa* and *S. aureus* reference and mutant strains.

**Strain**	**Description**	**Relevant property**	**References**
***P. aeruginosa***			
PA14	Clinical isolate UCBPP-PA14, Rif^R^	Prototypic reference strain	Rahme et al., 1995
PA14Δ*lasR/rhlR*	PA14 *lasR/rhlR*; Gen^R^, Tet^R^	Altered in quorum-sensing circuitry and all AQs^1^ production	Dekimpe and Déziel, 2009
PA14Δ*pqsA*	PA14 *pqsA*:TnphoA; Km^R^	Altered in HHQ^2^, PQS^3^, and HQNO^4^ production	Déziel et al., 2004
PA14Δ*lasA*	PA14 *lasA*:TnMrT7; Gen^R^	Deficient for the endopeptidase LasA	Liberati et al., 2006
***S. aureus***			
CF07-L	Clinical isolate	Prototypic reference strain	Mitchell et al., 2010
NRS149	Standard *agr* group II prototype	Prototypic reference strain	Ji et al., 1997; Lyon et al., 2000
NRS155	*agr*-null derivative of NRS149	Deficient for the Agr regulator	Lyon et al., 2000
Newbould	Reference isolate ATCC 29740	Prototypic reference strain	Moisan et al., 2006
Newbould*ΔsigB*	Newbould *sigB*:*emrA*; Erm^R^	Deficient for the alternative transcription factor SigB	Moisan et al., 2006
8325-4	Naturally deficient for *rsbU*	Reduced SigB activity	Mitchell et al., 2012
SH1000	Isogenic to 8325-4, but with a functional *rsbU* allele	Functional SigB activity	Mitchell et al., 2012
8325-4*ΔΔfnbAB*	8325-4 with Tet^R^ and Erm^R^ cassettes inserted in *fnbA* and *fnbB*; Tet^R^, Erm^R^	Reduced SigB activity; FnbA and FnbB absent	Mitchell et al., 2008

*^1^AQs, 2-alkyl-4-(1H)-quinolones; ^2^HHQ, 2-heptyl-4-hydroxyquinoline; ^3^PQS, *Pseudomonas* quinolone signal; ^4^HQNO, 2-heptyl-4-hydroxy quinoline *N*-oxide.*

### Growth Kinetics Experiments

The effect of *P. aeruginosa* isolates on *S. aureus* was investigated in growth kinetics experiments, similarly to those we previously described (Boulanger et al., 2015). Both *S. aureus* and *P. aeruginosa* were grown alone or in the presence of each other. Individual strains (10^5^–10^6^ CFU/ml) were used to inoculate CAMHB cultures. The cultures were incubated for 48 h with shaking (225 rpm) at 35°C. Samples were collected at 0, 2, 4, 6, 8, 24, and 48 h after the initial inoculation, serially diluted, and plated on TSA with 1 μg/ml of rifampicin (Clinical and Laboratory Standards Institute [CLSI], 2018) and on TSA with 10 μg/ml of polymyxin B (Clinical and Laboratory Standards Institute [CLSI], 2018) for selection of *P. aeruginosa* and *S. aureus* CFU, respectively. Bacterial counts were determined after a 24-h incubation at 35°C and confirmed after 48 h. Data were collected from at least three independent assays.

### Co-culture Petri Model

To visualize colony morphology and apparition of slow-growing colonies of *S. aureus* in the presence of *P. aeruginosa*, a co-culture Petri model was established. Approximately 10^5^CFU/ml of *S. aureus* was suspended in phosphate-buffered saline (PBS) and then spread on TSA plates. *P. aeruginosa* was suspended in PBS at a concentration of 10^7^–10^8^CFU/ml, then 10 μl of the suspension was spotted at the center of the *S. aureus* inoculated plates. Plates were incubated 24 h at 35°C, then areas of interest were photographed using a Leica M165 FC stereomicroscope (Leica, Concord, ON, Canada) with an objective of 0.63×. *S. aureus* showing a reduced colony size and a loss of pigmentation were considered slow-growing colonies and not strictly SCVs since they were not subcultured to see if they maintained their phenotype. Observations were collected from three independent experiments.

### Mouse Lung Mono- and Co-infection Model

The mouse model of pulmonary infection has been described previously (Mitchell et al., 2013) and was used here to investigate the extent of colonization by *S. aureus* and *P. aeruginosa in vivo* during mono and coinfections. Briefly, overnight bacterial cultures were used to inoculate fresh TSB at an *A*_600__*nm*_ of 0.1. Cultures were grown at 35°C with shaking (225 rpm) until the *A*_600__*nm*_ reached 0.6–0.8. Bacterial cells were then collected by centrifugation, washed, and suspended in PBS. Strains were suspended in 50 μl to concentrations required for infection: 2 × 10^6^ CFU for *S. aureus* and *P. aeruginosa* PA14, 2 × 10^7^ CFU for all the other clinical strains of *P. aeruginosa*. Such inocula were chosen because they were found to be an appropriate bacterial load to induce reproducible infections. For mixed infections, the quantity of total bacteria was equivalent to the sum of each inoculum used in mono infection. A sterilized 250-μl glass syringe (Hamilton Company, Reno, NV, United States) equipped with a bent feeding needle (Fine Science Tools, North Vancouver, BC, Canada) was used to infect CD-1 female mice (22–24 g, Charles River, Sherbrooke, QC, Canada). Animals were anesthetized with ketamine and xylazine and then, using an otoscope equipped with a speculum (model 21700, Welch Allyn, Mississauga, ON, Canada), the trachea was located, and the tip of the feeding needle was inserted. While maintaining the otoscope in place, 50 μl of the inoculum was instilled. After 24 h of infection, the animals were anesthetized, sacrificed, and then the lungs were harvested and homogenized using a Kinematica Polytron homogenizer 10-35 GT (Kinematica, Bohemia, NY, United States) in 1.5 ml of PBS. CFU were enumerated by serially diluting homogenates in PBS and plating on TSA with 1 μg/ml of rifampicin and on TSA with 10 μg/ml of polymyxin B, allowing selective growth of *P. aeruginosa* and *S. aureus*, respectively. Part of the homogenates was kept at −80°C until used for measurement of myeloperoxidase (MPO) activity (see below).

### MPO Activity

Assessment of inflammation and infiltration of neutrophils during mono or coinfections of mouse lung tissues were evaluated by quantification of MPO activity using the *o*-dianisidine-H_2_O_2_ method, as previously described (Côté-Gravel et al., 2016). Briefly, 10 μl of lung homogenate was mixed with a solution of *o*-dianisidine hydrochloride (167 μg/ml) (Sigma–Aldrich, Oakville, ON, Canada), 0.0005% H_2_O_2_ (Sigma–Aldrich, Oakville, ON, Canada), 50 mM hexade-cyltrimethylammonium bromide (CTAB) and 50 mM phosphate buffer at pH 6.0, in a 96-well plate. The *A*_460__*nm*_ was then measured at intervals of 15 s for 8 min and the maximum reaction rate was considered. One unit of MPO was defined as the quantity of enzyme degrading 1 μmol of H_2_O_2_/min at 25°C, with an absorption coefficient of 11.3 mM^–1^ cm^–1^ at 460 nm for *o*-dianisidine. MPO units were normalized according to the lung weight.

### RNA Isolation and RT-qPCR

Non-infected and infected (mono and coinfections) mouse lungs were homogenized on ice in 1 ml Trizol (Thermo Fisher Scientific, Rochester, NY, United States) using a Dounce tissue grinder, according to the manufacturer’s indications. RNA was extracted from homogenates using the RNeasy Mini Kit (Qiagen, Toronto, ON, Canada), following the manufacturer’s protocol. RNA integrity was verified by migration on 1% agarose gels and absence of residual DNA was confirmed by PCR with glyceraldehyde-3-phosphate dehydrogenase (GAPDH) primers and subsequent migration on 1.5% agarose gels. cDNA was obtained by reverse transcription from RNA using the 5× All-In-One RT MasterMix kit (Applied Biological Materials, Richmond, BC, Canada). Two microliters of cDNA was amplified with the SYBR Green JumpStart Taq ReadyMix (Sigma–Aldrich, Oakville, ON, Canada) by qPCR. GAPDH was used as an internal control gene for all samples, to relativize intercellular adhesion molecule 1 (ICAM-1) and integrin alpha-5 (ITGA-5) expression. The following primer DNA sequences were used:

ICAM-1 FWD 5′-GTTCCAGTATGACTCCACTCACGG-3′;
ICAM-1 REV 5′-CGGCCTCACCCCATTTGATGTTAG-3′;
ITGA-5 FWD 5′-TGTTTCAGGCTGCGCTGTGA-3′;
ITGA-5 REV 5′-CTGGCGGCTCAGTATCTCCTC-3′;
GAPDH FWD 5′-GTTCCAGTATGACTCCACTCACGG′3’;
GAPDH REV 5′-CGGCCTCACCCCATTTGATGTTAG-3′.

## Results

### Clinical Co-isolates Show Different Levels of Antagonism *in vitro*

**FIGURE 1:**
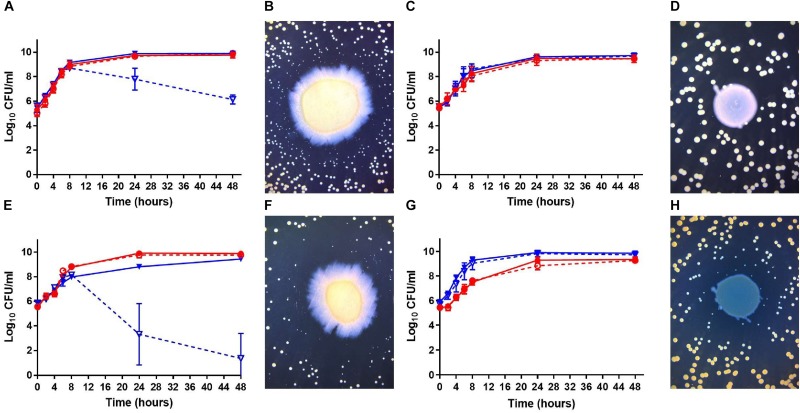
Growth kinetics and bacterial viability of *S. aureus* and *P. aeruginosa* in mono or co-cultures. **(A,C,E,G)** Bacteria were grown in broth cultures and viability is expressed in log_10_ CFU/ml. The CFUs were determined by plating sample dilutions on TSA supplemented with polymyxin B (*S. aureus*) or with rifampicin (*P. aeruginosa*). The solid dots (∙) and full red lines represent counts in *P. aeruginosa* mono-cultures; the open dots (∘) and dashed red lines, *P. aeruginosa* counts in co-cultures; the solid triangles (▼) and full blue lines, counts of *S. aureus* in mono-cultures; the open triangles (▽) and dashed blue lines, *S. aureus* counts in co-cultures. **(B,D,F,H)** *S. aureus* was plated on TSA and then a spot of *P. aeruginosa* was deposited in the center of the plate. The plates were photographed after 24 h of incubation. The *P. aeruginosa* and *S. aureus* pairs tested were PA14 and CF07-L **(A,B)**, PAC54A and CF54A-L **(C,D)**, PA14 and CF54A-L **(E,F)**, and PAC22A and CF22A-L **(G,H)**, respectively. The data shown were collected from at least three independent assays.

Using a collection of bacterial isolates from CF patients, Fugère et al. (2014) showed that co-isolated strains of *P. aeruginosa* and *S. aureus* interact differently *in vitro* than prototypical *P. aeruginosa* and *S. aureus* strains. Notably, when compared to prototypical strains, much less stimulation of *S. aureus* biofilm production was seen in presence of *P. aeruginosa* culture supernatants when co-isolates from CF patients were studied (Fugère et al., 2014). Interactions of co-isolates were thus further examined in the present work. Growth kinetics and viability of both species in co-cultures *in vitro* were measured. The prototypical strains PA14 and CF07-L, both extensively characterized in the scientific literature (Rahme et al., 1995; Mitchell et al., 2010), were considered as a suitable control pair for typical antagonistic interactions between these organisms. Figure 1 reports the types of interactions we observed. First, Figure 1A confirms the strong antagonism of PA14 over *S. aureus* CF07-L. Viability of *S. aureus* CF07-L was lowered by *P. aeruginosa* PA14 after 8 h of co-culture with viable counts dropping by 2.1 and 3.8 log_10_ CFU/mL at 24 and 48 h, respectively, compared to the counts of *S. aureus* in the mono-culture. Furthermore, co-culture on agar plates revealed formation of slow-growing colonies of *S. aureus* CF07-L around the large central colony of *P. aeruginosa* PA14 (Figure 1B), which is also typical of *P. aeruginosa* antagonism on *S. aureus* through the production of HQNO (Hoffman et al., 2006). We observed a similar antagonism by *P. aeruginosa* on *S. aureus* for the CF patient co-isolates PAC6B and CF6B-L, PAC39A and CF39A-L, and PAC112A and CF112A-L, respectively (Supplementary Figure S1), although the reduction of *S. aureus* CFU/mL counts at 48 h was less than that observed for CF07-L co-cultured with PA14 (i.e., 1.9, 2.1, and 3.6 log_10_, respectively). On the other hand, CF patient *P. aeruginosa* PAC54A did not affect the viability of its co-isolate *S. aureus* CF54A-L (Figure 1C) and no slow-growing colony of *S. aureus* and no growth inhibition was seen around *P. aeruginosa* on the agar plate (Figure 1D). When substituting *P. aeruginosa* PAC54A by PA14, a very strong antagonism toward *S. aureus* CF54A-L was observed (Figures 1E,F) showing that the lack of antagonism in Figures 1C,D was linked to *P. aeruginosa* PAC54A and not an insensitivity of *S. aureus* CF54A-L to *P. aeruginosa*. Interestingly, a different scenario was observed with the CF patient co-isolates *P. aeruginosa* PAC22A and *S. aureus* CF22A-L, where no effect on *S. aureus* viability was observed (Figure 1G) although slow-growing colonies of *S. aureus* appeared around the *P. aeruginosa* central colony (Figure 1H). In short, while *P. aeruginosa* PA14 displayed a strong antagonism on *S. aureus* CF07-L (or CF54A-L), the CF patient co-isolated *P. aeruginosa*–*S. aureus* pairs (i.e., PAC54A–CF54A-L, PAC22A–CF22A-L, PAC6B–CF6B-L, PAC39A–CF39A-L, PAC112A–CF112A-L) that we tested showed less or no antagonism in the co-culture *in vitro* models (broth and agar).

### *P. aeruginosa* Increases *S. aureus* Colonization in a Mouse Lung Infection Model, Regardless of Their Type of Interactions *in vitro*

**FIGURE 2:**
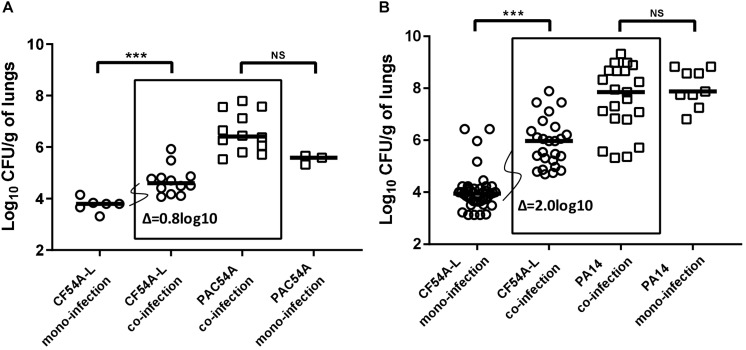
Mouse pulmonary mono or co-infections with *S. aureus* and *P. aeruginosa.* CFUs were determined 24 h post-infection by plating lung homogenates on TSA supplemented with polymyxin B (*S. aureus*) or with rifampicin (*P. aeruginosa*). CFUs obtained from co-infections are presented in boxes. For co-infections, the starting inoculum was equivalent to the sum of each inoculum used in mono-infections. The pairs tested were *P. aeruginosa* PAC54A and *S. aureus* CF54A-L **(A)**, and *P. aeruginosa* PA14 and *S. aureus* CF54A-L **(B)**. Each symbol represents the lungs of one mouse. The median for each group is indicated by the horizontal bar. Statistical differences between the median log_10_ CFU per gram of lungs for mono and co-infections for both *P. aeruginosa* and *S. aureus* were determined with a Mann–Whitney test: NS, not statistically significant, *p* > 0.05; ^∗∗∗^*p* < 0.001.

Considering that we have observed that some *S. aureus*–*P. aeruginosa* clinical co-isolates do not necessarily interact as prototypical strains, namely by a lack or a lesser degree of antagonism *in vitro*, we wanted to examine if such *in vitro* observations correlated with the outcome of co-infections *in vivo* using a mouse pulmonary infection model. The level of bacterial colonization of the lungs following a mono-infection (either by *S. aureus* or by *P. aeruginosa*) was compared to the level of colonization obtained after a 24-h co-infection by both pathogens by measuring species-specific CFU counts. Several *S. aureus*–*P. aeruginosa* pairs were tested. *P. aeruginosa* PAC54A, a clinical strain showing no antagonism *in vitro* toward its co-isolate *S. aureus* CF54A-L (Figures 1C,D), promoted *S. aureus* lung colonization by a median increase of 0.8 log_10_ (Figure 2A) in comparison to the mono-infection of CF54A-L. Besides, strains *P. aeruginosa* PA14 and *S. aureus* CF07-L displayed antagonism in the *in vitro* models described earlier (Figures 1A,B) and were considered again as a typical control pair. Surprisingly, the antagonism observed *in vitro* did not translate *in vivo* and, on the contrary, cooperation was observed as *P. aeruginosa* PA14 increased *S. aureus* CF07-L CFU counts compared to the mono-infection (a median increase of 1.7 log_10_, Supplementary Figure S2). Due to this unexpected *in vivo* cooperation from a bacterial pair showing antagonism *in vitro*, PA14 co-infection was tested again, but this time with *S. aureus* CF54A-L, which was even more strongly affected by *P. aeruginosa* PA14 *in vitro* (Figures 1E,F). Once again, *S. aureus* CF54A-L colonization was enhanced by the presence of *P. aeruginosa* PA14 despite strong antagonism *in vitro* (2.0 log_10_ median increase compared to the mono-infection, Figure 2B). Noteworthy, in all cases, *P. aeruginosa* colonization was never promoted by the presence of *S. aureus* (comparison of *P. aeruginosa* mono and co-infections, not statistically significant, Figures 2A,B and Supplementary Figure S2). In summary, for all the tested strains, *P. aeruginosa* promotes *S. aureus* lung colonization, even if it is antagonistic *in vitro*.

### Searching for *P. aeruginosa* Virulence-Associated Factors Helping *S. aureus* Colonization

**FIGURE 3:**
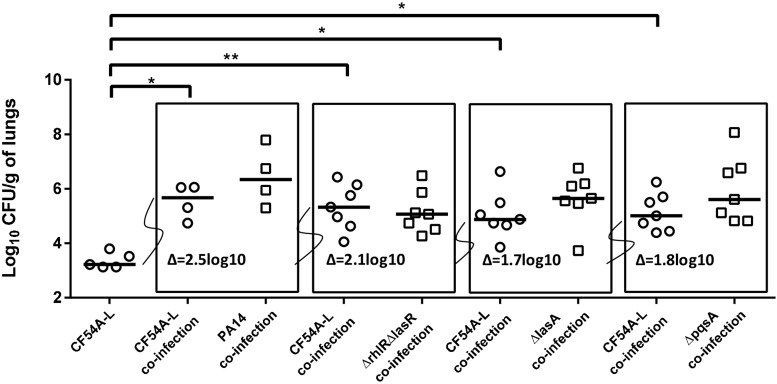
Mouse pulmonary mono or co-infections with *S. aureus* CF54A-L and *P. aeruginosa* PA14 and mutants. CFUs were determined 24 h post-infection by plating lung homogenates on TSA supplemented with polymyxin B (*S. aureus*) or with rifampicin (*P. aeruginosa*). CFUs obtained from co-infections are presented in boxes. For co-infections, the starting inoculum was equivalent to the sum of each inoculum used in mono-infection. The pairs tested were *S. aureus* CF54A-L and *P. aeruginosa* PA14; CF54A-L and PA14Δr*hlR*Δ*lasR*; CF54A-L and PA14Δ*lasA*; CF54A-L and PA14Δ*pqsA*. Each symbol represents the lungs of one mouse. The median for each group is indicated by the horizontal bar. Statistical differences between the median log_10_ CFU per gram of lungs for *S. aureus* CF54A-L mono and co-infections were determined with a Mann–Whitney test: NS, not statistically significant, *p* > 0.05; ^∗^*p* < 0.05; ^∗∗^*p* < 0.01.

In an attempt to determine if a specific *P. aeruginosa* virulence-associated factor was responsible for the promotion of *S. aureus* lung colonization, PA14 mutants displaying different alterations, ranging from global virulence regulators to specific virulence factors (Table 1), were tested in the co-infection assay (all used at an inoculum of ∼2 × 10^6^ CFU). *S. aureus* colonization was still improved when co-infecting with any of the PA14 mutants tested (PA14Δ*rhlR*Δ*lasR*, 2.1 log_10_ median increase; PA14Δ*lasA*, 1.7 log_10_ increase; PA14Δ*pqsA*, 1.8 log_10_ increase) but the promotion was less than that observed with wild-type PA14 (2.5 log_10_ increase) (Figure 3). Therefore, none of the specific *P. aeruginosa* factors tested here could be identified as crucial for helping *S. aureus* colonization using this approach.

### Searching for *S. aureus* Virulence-Associated Factors Promoting Its Own Colonization During Co-infection

**FIGURE 4:**
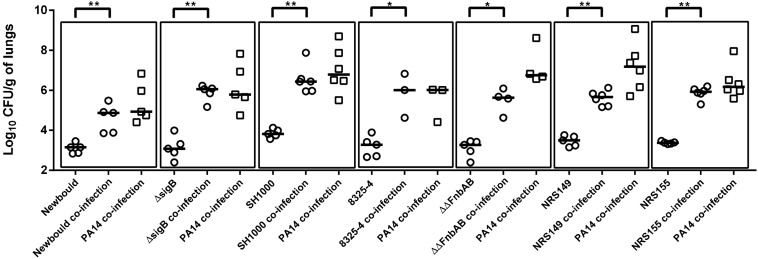
Mono or mixed mouse pulmonary infections with *S. aureus* virulence mutants and *P. aeruginosa.* CFUs were determined 24 h post-infection by plating lung homogenates on TSA supplemented with polymyxin B (*S. aureus*) or with rifampicin (*P. aeruginosa*). CFUs obtained from co-infections are presented in boxes. For co-infections, the starting inoculum was equivalent to the sum of each inoculum used in mono-infections. The pairs tested were *S. aureus* Newbould and *P. aeruginosa* PA14; NewbouldΔsigB and PA14; SH1000 and PA14; 8325-4 and PA14; 8325-4ΔΔfnbAB and PA14; NRS149 and PA14; NRS155 and PA14. Each symbol represents the lungs of one mouse. The median for each group is indicated by the horizontal bar. Statistical differences between the median log_10_ CFU per gram of lungs for every *S. aureus* mono and co-infection were determined with a Mann–Whitney test: NS, not statistically significant, *p* > 0.05; ^∗^*p* < 0.05; ^∗∗^*p* < 0.01.

Since it was determined that *P. aeruginosa* mutants showing some attenuation in virulence still improved *S. aureus* colonization proportionally to their own colonization, the contribution of *S. aureus* in this phenomenon was also examined using a similar approach. Co-infections were performed using *P. aeruginosa* PA14 and various *S. aureus* mutants (all used at an inoculum of 2 × 10^6^ CFU). The contribution of the global regulators SigB and Agr was investigated since they both are major virulence regulators in SCVs and wild-type strains (Novick and Geisinger, 2008; Mitchell et al., 2013). Therefore, given their large influence over *S. aureus* virulence, it seemed plausible they could be implicated in the improved colonization of *S. aureus* in presence of *P. aeruginosa* and could eventually lead to the identification of a precise responsible *S. aureus* factor. However, when tested in the lung co-infection model, NewbouldΔ*sigB* colonization was increased by the presence of PA14, in a similar manner to that observed for the *S. aureus* wild-type counterpart Newbould (Figure 4). Similarly, 8325-4 is a *S. aureus* strain naturally deficient in SigB activity because of its defective *rsbU* alleles. Strain 8325-4 was thus compared to SH1000, an isogenic strain with a restored *rsbU* allele (O’Neill, 2010). Again, PA14 positively affected colonization of either *S. aureus* strains (8325-4 and SH1000) compared to *S. aureus* mono-infections. Next, using the same strain background (8325-4) we investigated a mutant for the fibronectin-binding proteins A and B, which are known multi-purpose adhesins for *S. aureus* (Josse et al., 2017). Despite the absence of FnbAB, Figure 4 shows that *P. aeruginosa* still helps *S. aureus* lung colonization. The role of the *S. aureus* Agr system was then examined. Agr is a global regulator of *S. aureus* virulence that influences expression of exoproducts and surface proteins. Nevertheless, NRS155, an *agr*-null derivative of NRS149, was still significantly helped in its lung colonization by *P. aeruginosa* PA14. In conclusion, neither the alternative transcription factor SigB, the adhesins FnbAB, or the Agr regulator seem to contribute to the mechanism by which *P. aeruginosa* helps the colonization of the lungs by *S. aureus*.

### *P. aeruginosa* Improves *S. aureus* Colonization in a Dose-Dependent Manner

**FIGURE 5:**
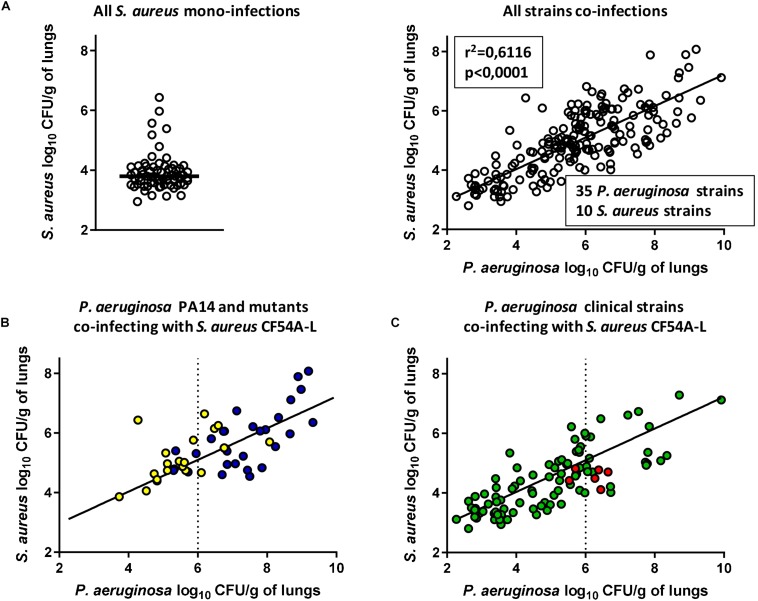
Compilation of mixed mouse pulmonary infections with *S. aureus* and *P. aeruginosa.* CFUs were determined 24 h post-infection by plating lung homogenates on TSA supplemented with polymyxin B (*S. aureus*) or with rifampicin (*P. aeruginosa*). For co-infections, the starting inoculum was equivalent to the sum of each inoculum used in mono-infections. The strains tested in “All *S. aureus* mono-infections” were *S. aureus* CF07-L, CF54A-L, and CF112A-L at an inoculum of 2 × 10^6^CFU **(A, left)**. In “All strains co-infections,” the strains tested were CF07-L, CF54A-L, CF112A-L, and every *S. aureus* strain indicated in Table 1, co-infecting with *P. aeruginosa* PA14, PAC54A, and PAC112A, all PA14 mutants indicated in Table 1 and 29 clinical isolates indicated in Supplementary Table S1 **(A, right)**. In “*P. aeruginosa* PA14 and mutants co-infecting with *S. aureus* CF54A-L,” the strains tested were CF54A-L co-infecting with PA14 and its associated virulence mutants at an inoculum of 2 × 10^6^ CFU **(B)**. In “*P. aeruginosa* clinical strains co-infecting with *S. aureus* CF54A-L,” the strains tested were CF54A-L co-infecting with every *P. aeruginosa* clinical strain, including PAC54A, at an inoculum of 2 × 10^7^ CFU **(C)**. Each symbol represents the lungs of one mouse. Statistical significance of the trendline of all strain co-infections was determined with a linear regression test. Blue dots represent co-infections with PA14; yellow dots represent co-infections with PA14 virulence mutants; green dots represent co-infections with clinical *P. aeruginosa* strains; and red dots represent co-infections with PAC54A.

Following the previous results, we formulated the hypothesis that during a co-infection, the higher the titer of *P. aeruginosa* in the lungs, the more *S. aureus* colonization would be enhanced. To test this hypothesis, the pulmonary co-infection model was employed using a collection of clinical and mutant strains. We used 35 *P. aeruginosa* strains in combination with 10 *S. aureus* strains and different inoculum sizes were used for some strains for a total of 200 co-infections. For each experimental co-infection, the CFU counts in the lungs for *P. aeruginosa* and for *S. aureus* were determined after 24 h and plotted in Figure 5A, which includes results using the *P. aeruginosa* PA14 mutants and *S. aureus* mutants of Figures 3, 4, respectively. Based on Figure 5A, it was clear that higher CFU counts of *P. aeruginosa* increased the colonization of *S. aureus* in a dose-dependent manner (Figure 5A, right panel, linear regression *R*^2^ = 0.6116, *p* < 0.0001). Figure 5A also shows that most of the *S. aureus* strains used in this model as mono-infections do not colonize the lungs very efficiently (median log_10_ CFU/g of lung of 3.8, Figure 5A, left panel). This shows once again that higher titers of *P. aeruginosa* in infected lungs drive upward and not downward the colonization potential of *S. aureus*.

To better understand the correlation between *P. aeruginosa* and *S. aureus* colonization, the data from Figure 5A were analyzed separately for different *P. aeruginosa* subgroups. First, results for *P. aeruginosa* PA14 and its mutants (all used at an inoculum of ∼2.0 × 10^6^ CFU) in co-infection with *S. aureus* strain CF54A-L are shown in Figure 5B. While infection by the mutant strains was less productive than that parent PA14 infections (generally yielding less than 10^7^ CFU/g of lungs), colonization of *S. aureus* was still proportional to that of *P. aeruginosa*.

Data for *P. aeruginosa* clinical strains (all used at an inoculum of ∼2 × 10^7^ CFU) in co-infection with *S. aureus* strain CF54A-L (shown to be susceptible to *P. aeruginosa* antagonism *in vitro*, Figures 1E,F) demonstrated once again the same correlation (Figure 5C). *P. aeruginosa* strain PAC54A (the non-antagonistic strain *in vitro*, Figures 1C,D) also fitted the linear regression (Figure 5C, red symbols) although this *P. aeruginosa* strain was less productive and accordingly, *S. aureus* colonization was less abundant. Similar to the *P. aeruginosa* PA14 mutants deficient in a variety of virulence-associated products (Figures 3, 5B), most *P. aeruginosa* clinical strains yielded less than 10^7^ CFU/g of lungs (Figure 5C). Data for specific *S. aureus* strains and mutants, all in co-infection with *P. aeruginosa* PA14, are plotted in Supplementary Figure S3. No particular *S. aureus* mutant seemed to diverge from the trend described above.

Overall, each *P. aeruginosa* strain tested enhanced *S. aureus* colonization proportionally to their own ability to infect the lungs. *P. aeruginosa* clinical strains generally yielded lower levels of lung colonization compared to the prototypic strain PA14.

### The Contribution of *P. aeruginosa* to *S. aureus* Colonization Is Independent of MPO Induction

**FIGURE 6:**
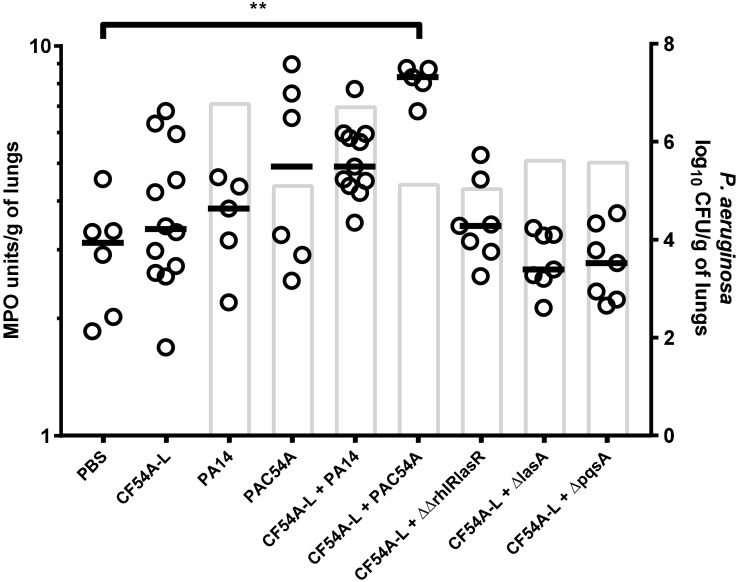
MPO activity of lungs mono or co-infected with *S. aureus* and *P. aeruginosa.* MPO units were determined using an enzymatic assay. CFUs were determined 24 h post-infection by plating lung homogenates on TSA supplemented with polymyxin B (*S. aureus*) or with rifampicin (*P. aeruginosa*). For co-infections, the starting inoculum was equivalent to the sum of each inoculum used in mono-infections. The strains tested were *S. aureus* CF54A-L; *P. aeruginosa* PA14, PA14Δ*rhlR*Δ*lasR*, PA14Δ*lasA*, PA14Δ*pqsA*, and PAC54A. The median for MPO activity in each group is indicated by the horizontal bar, while open dots represent the MPO activity for an individual lung. Columns indicate the median *P. aeruginosa* colonization for each group. Statistical differences between the median MPO activity for PBS control and the other groups were determined with a Kruskal–Wallis test: ^∗∗^*p* < 0.01

Since no *P. aeruginosa* or *S. aureus* virulence factors could be identified as an essential determinant in the mechanism by which *P. aeruginosa* stimulates *S. aureus* colonization, other possible causes possibly involved in the beneficial effect of *P. aeruginosa* on the colonization of *S. aureus* were investigated. It was thus hypothesized that *P. aeruginosa* could exacerbate a pro-inflammatory response, which could help *S. aureus* colonization. MPO activity was therefore measured for a series of co-infections in the mouse since it was recently found to be a good indicator of inflammation (Côté-Gravel et al., 2016). *S. aureus* CF54A-L was used in all co-infections together with a variety of *P. aeruginosa* strains showing different levels of virulence and colonization. All *P. aeruginosa* strains were compared, using an inoculum of ∼2 × 10^6^ CFU for PA14 and mutants and an inoculum of ∼2 × 10^7^ CFU for PAC54A and *P. aeruginosa* clinical strains. Interestingly, only the co-infection of *P. aeruginosa* PAC54A and *S. aureus* CF54A-L resulted in a significantly higher MPO score (vs. the PBS control) even though the level of colonization for both species in that co-infection was much less than that achieved by the PA14–CF54A-L pair (Figure 6). This clearly indicates that a high colonization of *P. aeruginosa* does not necessarily translate into a high MPO score and that the level of MPO does not correlate with the ability of *P. aeruginosa* to promote *S. aureus* colonization. Also inversely, low MPO induction (as seen with the PA14 co-infections) does not better promote *S. aureus* colonization. Hence, the level of inflammation, as inferred by MPO production elicited by *P. aeruginosa* or the co-infection, does not seem to be involved in the mechanism by which *P. aeruginosa* helps *S. aureus* colonization.

### *P. aeruginosa* Induces Overexpression of Known *S. aureus* Cell Surface Receptors

**FIGURE 7:**
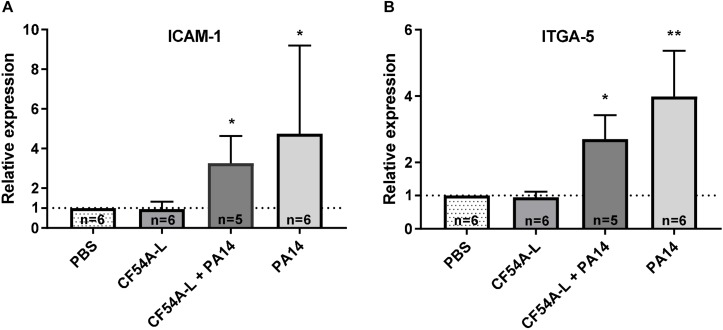
Relative expression of cellular ICAM-1 **(A)** and ITGA-5 **(B)** genes in lung tissues during mono or co-infections with *S. aureus* CF54A-L and *P. aeruginosa* PA14. Total RNA was extracted from lung tissues 24 h post-infection. Expression was quantified by RT-qPCR and normalized to the PBS control. GAPDH expression was used as an internal control for each individual sample. Quantity of biological replicates (*n*) is indicated for each group. Statistical differences between the relative expression of both genes in the infection groups compared to the PBS control group were determined with a Kruskal–Wallis test. ^∗^*p* < 0.05; ^∗∗^*p* < 0.01.

Since neither virulence-associated factors nor the level of inflammation could explain the promotion of *S. aureus* colonization by *P. aeruginosa*, we looked for other possibilities inspired by knowledge surrounding bacterial and viral co-infections. Indeed, it has been shown that rhinoviruses can promote cellular ICAM-1 and integrin α_5_β_1_ expression (Passariello et al., 2006), which have been previously described as cell surface receptors for *S. aureus*. Therefore, we verified by RT-qPCR if *P. aeruginosa* could induce a similar effect on lung tissue during infection. To this end, PA14 effect was investigated, as it was shown to be a strong inducer of *S. aureus* colonization. Figure 7A shows that a *P. aeruginosa* PA14 mono- or co-infection with *S. aureus* CF54A-L induced a significant increase in the expression of ICAM-1 in comparison to the PBS control. In addition, the expression of ITGA-5, which associates with the β_1_ subunit to form the α_5_β_1_ integrin, was also enhanced by the co-infection (Figure 7B), while on the other hand, a mono-infection by *S. aureus* CF54A-L did not change the level of expression of either ICAM-1 or ITGA-5. For each biological sample tested, a GAPDH internal control was added to ensure adequate RNA integrity and for relative quantification of gene expression levels. At least five biological replicates were produced for each infection group. Hence, PA14, during both mono- and co-infections, appeared to significantly stimulate the expression of these two cellular genes, which might in turn contribute to *S. aureus* colonization.

## Discussion

This study aimed to gain a better understanding of the interactions between *P. aeruginosa* and *S. aureus*, which are allegedly antagonistic to each other *in vitro*, although they are commonly co-isolated from chronically infected CF patients. Also, we previously demonstrated that clinical *P. aeruginosa* and *S. aureus* co-isolates from adult CF patients do not necessarily display the same properties as prototypical antagonistic strains *in vitro*. One such a discrepancy between prototypic and clinical isolates properties was the relative ability of *P. aeruginosa* to induce biofilm production by *S. aureus in vitro*, a phenomenon strongly correlated to the quantity of HQNO produced by the clinical isolates of *P. aeruginosa* (Fugère et al., 2014). Studying further *P. aeruginosa*–*S. aureus* co-isolates should provide the necessary data to close the gap between the seemingly opposite *in vitro* and *in vivo* behaviors. Coherently with data displayed in our previous study, we demonstrated here that *P. aeruginosa* and *S. aureus* co-isolates do not always antagonize each other *in vitro*. Such results indicate that *P. aeruginosa* may become less aggressive toward *S. aureus* in the CF lung environment. This may either be the result of an adaptation toward *S. aureus* or to the hostile host environment and inflammatory response. By reducing its production of alkyl-quinolones and other QS factors, *P. aeruginosa* is less likely to trigger an intense immune response. At the same time, with a reduced production of HQNO and alkyl-quinolones, *P. aeruginosa* dampens its inhibitory effect against *S. aureus* (Hoffman et al., 2006). This could be the case for *P. aeruginosa* PAC54A, which produces very little HQNO and other QS factors compared to PA14 (Fugère et al., 2014; Supplementary Material), and which displays no antagonism toward its *S. aureus* co-isolate CF54A-L (Figures 1C,D). Besides, PAC22A, which produces a similar amount of HQNO compared to that of PA14 (Fugère et al., 2014; Supplementary Material), also did not reduce the viability of its co-isolate CF22A-L although it still stimulated the formation of slow-growing colonies (Figures 1G,H). This shows the complex interactions that can exist between these bacterial species. Overall, our findings establish that contrary to the long-held belief, *P. aeruginosa* does not always antagonize *S. aureus in vitro* and the effect of *P. aeruginosa* on *S. aureus* viability and phenotypes can vary.

To our knowledge, very few studies have specifically examined the interaction between *S. aureus* and *P. aeruginosa* strains co-isolated from CF patients. Since co-isolates do not necessarily interact as prototypical strains *in vitro*, we hypothesized that co-isolates could also behave differently *in vivo*, which in turn could provide some explanation on why *S. aureus* and *P. aeruginosa* are frequently co-isolated from the CF lung. According to our findings, it seems that the nature of the interaction between *P. aeruginosa* and *S. aureus in vitro* is not a good indicator of the outcome of a co-infection *in vivo*. Unexpectedly, the success of *S. aureus* colonization during co-infection correlated with the extent of *P. aeruginosa* colonization in the lungs, irrespective of the type of interactions (indifference or antagonism) between these two microorganisms *in vitro*. Also, using a series of *P. aeruginosa* PA14 mutants, none of the tested virulence-associated factor in this current study was specifically identified as responsible for the promotion of *S. aureus* colonization during co-infection. Still, as the ability of the *P. aeruginosa* clinical strains to infect and promote *S. aureus* co-colonization ranged from low to high, it would be important to identify *P. aeruginosa* properties that may be conserved among the best colonizers, and therefore the best inducer of *S. aureus* colonization. Whole-genome sequencing is currently underway to compare *P. aeruginosa* isolates that are “low” and “high” inducers of *S. aureus* colonization to identify *P. aeruginosa* factors or mutations that best profit *S. aureus in vivo*.

The contribution of *S. aureus* key virulence regulators or effectors to the outcome of *P. aeruginosa*–*S. aureus* co-infections was also investigated in this study. It is known that *S. aureus* can adopt the SCV phenotype in the presence of prototypic *P. aeruginosa* strains which produce HQNO (Hoffman et al., 2006). SCVs are proficient in the invasion of non-professional phagocytic cells, which in turn helps them to evade the immune system (Mitchell et al., 2011). It was therefore possible that *P. aeruginosa* enhances *S. aureus* colonization *in vivo* by inducing SCV-like properties. Since the alternative transcription factor SigB is a dominant regulator of virulence in SCVs (Mitchell et al., 2013), we tested the colonization ability of two SigB deleted or altered mutants in the presence of *P. aeruginosa*. Using such an approach, we were not able to demonstrate a contribution of *S. aureus* SigB to the outcome of co-infection in mice. However, since the murine infection model was acute and not chronic, it is possible that in such conditions, induction of SCVs, cellular invasion, and intracellular replication might have been less significant. Alternatively, another hypothesis was that *P. aeruginosa* might affect and positively upregulate virulence in *S. aureus* but again, an *agr* mutant was not altered in its ability to co-colonize the lung with *P. aeruginosa* even though Agr is an important virulence activator in prototypic *S. aureus* strains (Novick and Geisinger, 2008).

These results indicate that *P. aeruginosa* is probably not directly affecting *S. aureus* virulence. Since *P. aeruginosa* inhibits or is at best indifferent toward *S. aureus in vitro*, we then can only infer that the environment must be modified by *P. aeruginosa* in a way that it promotes *S. aureus* colonization *in vivo*. *P. aeruginosa* can induce inflammation with a panel of different virulence factors (Wieland et al., 2002; Lin and Kazmierczak, 2017). While inflammation is necessary for controlling bacterial infections, an over-stimulated inflammatory response can provoke host tissue damage and alter bacterial clearance (Lin and Kazmierczak, 2017). Also, we have shown that activation of NF-κB by LPS and TNF-α increases *S. aureus* invasion of pulmonary cells cultured *in vitro* (Mitchell et al., 2011). It is therefore possible that inflammation provoked by *P. aeruginosa* can contribute to *S. aureus* colonization. We investigated this possibility by quantifying MPO, as a marker for inflammation. However, we showed here that the *P. aeruginosa* strain inducing the most MPO production in the mice lungs was PAC54A although it was not as efficient as the prototypic strain PA14 at promoting *S. aureus* infection. Therefore, it was not possible to associate the level of inflammation (based on MPO activity) with the ability of *P. aeruginosa* to enhance *S. aureus* colonization.

It is now well-recognized that viral infections of the respiratory tract can enhance the possibility of bacterial superinfections and this research topic has been reviewed (Fedy Morgene et al., 2018). Rhinoviruses have already been identified as microorganisms able to promote *S. aureus* colonization *in vivo*. Explicitly, rhinoviruses increase *S. aureus* colonization by a mechanism involving the release of IL-6, IL-8, and the overexpression of ICAM-1 (Passariello et al., 2006). Moreover, rhinoviruses also upregulate integrin α_5_β_1_ transcription (Kim et al., 2015). This integrin is one of the main pathways by which *S. aureus* can invade non-professional phagocytic cells (Josse et al., 2017). Hence, based on such a precedent for *in vivo* cooperation between two microorganisms, we investigated the expression of ICAM-1 and ITGA-5 (a marker for integrin α_5_β_1_) in mouse lung tissue infected by *P. aeruginosa* and *S. aureus*. Interestingly, *P. aeruginosa* mono- or co-infections could indeed increase expression of both host cell components, whereas a mono-infection with *S. aureus* did not (Figure 7). ICAM-1 is responsible for the transmigration of leukocytes during an infection, which occurs through the endothelium to the site of the lesions (Springer, 1990). *P. aeruginosa* induces its overexpression, possibly through tissue damage and the inflammatory process. Likewise, Gram negative LPS was shown to induce expression of ITGA-5 (Roman et al., 2004; Sampaio et al., 2010). It is thus plausible that *S. aureus* might benefit from these transcriptional changes to adhere to host cells and increase its colonization of the lung tissue when *P. aeruginosa* is present. Moreover, while we found that ITGA-5 and ICAM-1 expression was upregulated in lung cells during *P. aeruginosa* infection, many other transcriptional changes could definitely occur. Conducting a dual RNAseq on either co-infected lungs (or on a mixed infection in a cell culture model) would lead to a better understanding of the changes of the host cells occurring in presence of *P. aeruginosa* and *S. aureus* (Westermann et al., 2017).

*Staphylococcus aureus* possesses a wide variety of adhesins (Fedy Morgene et al., 2018; Foster, 2019). *S. aureus* FnBPs are bacterial adhesins known to interact with the integrin α_5_β_1_ through fibronectin (Mitchell et al., 2008). Since we showed that colonization of *S. aureus* lacking FnBPs is still promoted by co-infection with *P. aeruginosa*, such a *S. aureus* mutant must have other means to interact with the host cells. For example, the *S. aureus* protein EAP, which also binds fibronectin (Sinha and Fraunholz, 2010), can perhaps compensate for a lack of FnBPs. Also, *S. aureus* teichoic acids were shown to contribute to binding to endothelial cells (Weidenmaier et al., 2005). Consequently, a *S. aureus* strain lacking FnBPs should still adhere to host cells and colonize tissues (Josse et al., 2017). Furthermore, ICAM-1 and integrin α_5_β_1_ can both individually allow *S. aureus* host cell binding (Sinha et al., 1999; Passariello et al., 2006), and according to the mouse ENCODE transcriptome data, ICAM-1 is up to 10 times more prevalent than ITGA-5 in mice lungs (Yue et al., 2014). Likewise, ICAM-1 is overexpressed in comparison to ITGA-5 in the human lungs (Fagerberg et al., 2014). Hence, prototypic *S. aureus* and the FnBPs mutant may both have predominantly interacted with ICAM-1 to permit colonization in the murine lung infection model.

By using an acute (24 h) lung infection model, we were able to examine a large panel of bacterial strains, including mutants and clinical isolates. The model rapidly granted us robust results and increased statistical power. Not only was it possible to establish a model that helped us to gain some insights that could possibly explain the often worse clinical prognosis of *S. aureus*–*P. aeruginosa* coinfections, but we could also test many different hypotheses regarding the mechanism by which *P. aeruginosa* improves *S. aureus* colonization. However, infections afflicting CF patients are mostly chronic, therefore a chronic lung infection model would probably better mimic the real clinical conditions. Future experiments should be conducted using a chronic infection model to confirm results obtained in the present study.

Overall, we showed that *P. aeruginosa* promotes *S. aureus* colonization in a dose-dependent manner *in vivo*. The mechanism could involve inflammation and induction of ICAM-1 and ITGA-5, which would allow a better adhesion and colonization of *S. aureus*. Further experiments are required to identify precisely how *S. aureus* colonization benefits from *P. aeruginosa* impact on the lungs.

## Data Availability Statement

All datasets generated for this study are included in the article/Supplementary Material.

## Ethics Statement

The animal study was reviewed and approved by the institutional ethics committee on animal experimentation of the Faculté des Sciences of Université de Sherbrooke.

## Author Contributions

GM did the growth kinetics experiments *in vitro*, co-cultured petri dish assays, lung infections in mice, MPO extraction and quantification, verified ICAM-1 and ITGA-5 expression by qPCR, and wrote the article. J-PL contributed to the growth kinetics experiments. EB worked to develop the lung infection model. EF supervised the bacteria strains isolation. AC supervised the bacteria strains isolation and clinical evaluation of CF patients. FM contributed to the working plan and manuscript writing, and is the corresponding author.

## Conflict of Interest

The authors declare that the research was conducted in the absence of any commercial or financial relationships that could be construed as a potential conflict of interest.
