# Supplementary material for: Despite Antagonism in vitro, Pseudomonas aeruginosa Enhances Staphylococcus aureus Colonization in a Murine Lung Infection Model
Source: Front Microbiol. 2019 Dec 13;10:2880. doi: 10.3389/fmicb.2019.02880 (PMC6923662; doi:10.3389/fmicb.2019.02880)
Supplement: Supplementary file 1 [file Data_Sheet_1.pdf]

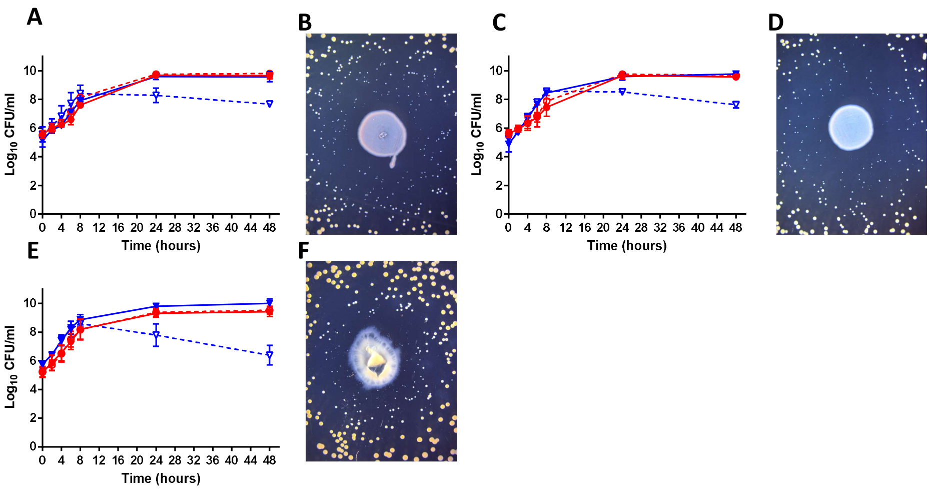

Figure S1 Growth kinetics and bacterial viability of *S. aureus* and *P. aeruginosa* in mono or co-cultures. (**A, C, and E**) Bacteria were grown in broth cultures and viability is expressed in $\log_{10}$ CFU/ml. The CFU were determined by plating sample dilutions on TSA supplemented with polymyxin B (*S. aureus*) or with rifampicin (*P. aeruginosa*). The solid dots (●) and full red lines represent counts in *P. aeruginosa* mono-cultures; the open dots (○) and dashed red lines, *P. aeruginosa* counts in co-cultures; the solid triangles (▼) and full blue lines, counts of *S. aureus* in mono-cultures; the open triangles (▽) and dashed blue lines, *S. aureus* counts in co-cultures. (**B, D and F**) The Petri assay was realized by plating *S. aureus* on TSA and applying a spot of *P. aeruginosa* in the center of the plate. The plates were photographed after 24h of incubation. The *P. aeruginosa* and *S. aureus* pairs tested were PA6B and CF6B-L (**A and B**), PAC39A and CF39A-L (**C and D**), and PAC112A and CF112A-L (**E and F**), respectively.

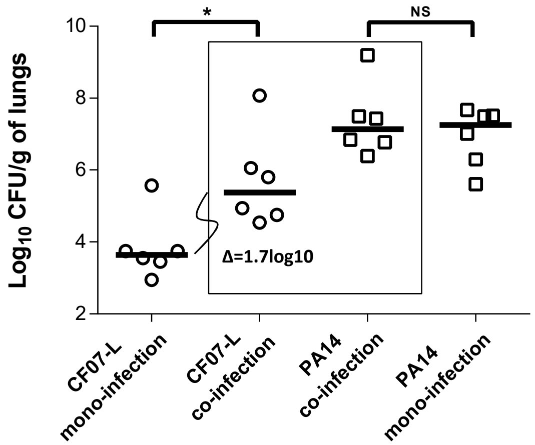

Figure S2 Mouse pulmonary mono or co-infections with *S. aureus* and *P. aeruginosa*. CFUs were determined 24 h post-infection by plating lungs homogenates on TSA supplemented with polymyxin B (*S. aureus*) or with rifampicin (*P. aeruginosa*). CFUs retrieved from co-infections are indicated by boxes. For co-infections, the starting inoculum was equivalent to the sum of each inoculum used in mono-infections. The pairs tested were *P. aeruginosa* PA14 and *S. aureus* CF07-L. The median for each group is indicated by the horizontal bar. Statistical differences between the median $\log_{10}$ CFU per gram of lungs for mono and co-infections for both *P. aeruginosa* and *S. aureus* were determined with a Mann-Whitney test : NS, not statistically significant, $p > 0.05$; *, $p < 0.05$.

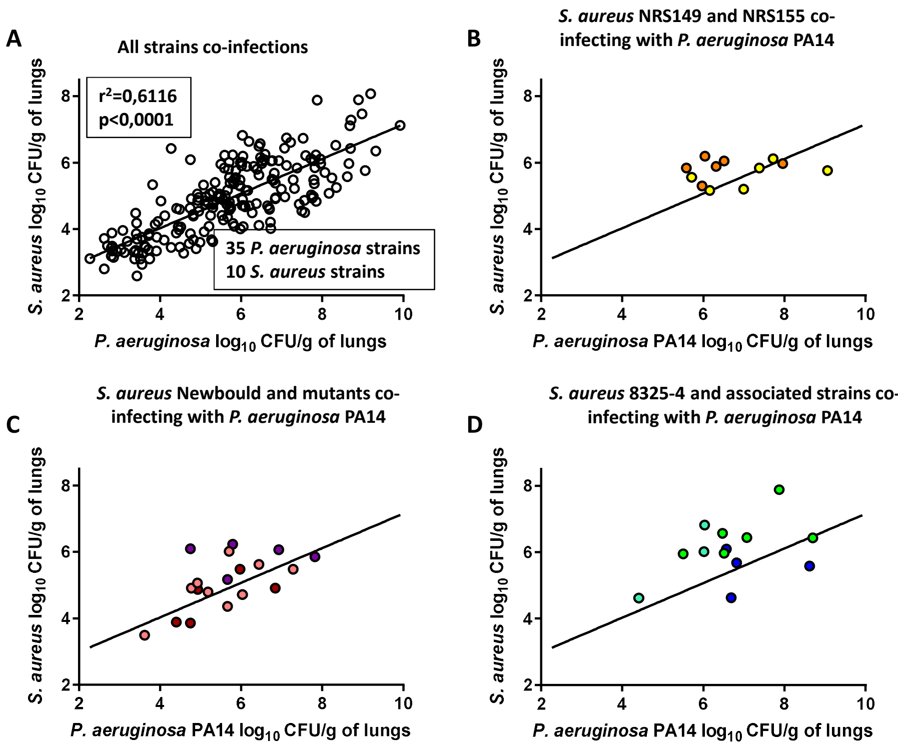

Figure S3 Compilation of mixed mouse pulmonary infections with *S. aureus* and *P. aeruginosa.* CFUs were determined 24 h post-infection by plating lungs homogenates on TSA supplemented with polymyxin B (*S. aureus*) or with rifampicin (*P. aeruginosa*). For co-infections, the starting inoculum was equivalent to the sum of each inoculum used in mono-infections. The strains tested in "All strains co-infections" were *S. aureus* CF07-L, CF54A-L, CF112A-L and every *S. aureus* strains indicated in Table 1, co-infecting with *P. aeruginosa* PA14, PAC54A, PAC112A, all PA14 mutants indicated in Table 1 and 29 clinical isolates (**A**). In "*S. aureus* NRS149 and NRS155 co-infecting with *P. aeruginosa* PA14", the strains tested were NRS149 and NRS155 co-infecting with PA14, at an inoculum of $2x10^6$ CFU (**B**). In "*S. aureus* Newbould and mutants co-infecting with *P. aeruginosa* PA14", the strains tested were Newbould, NewbouldΔ*sigB* and NewbouldΔ*hemB* co-infecting with PA14, at an inoculum of $2x10^6$ CFU (**C**). In "*S. aureus* 8325-4 and associated strains co-infecting with *P. aeruginosa* PA14", the strains tested were SH1000, 8325-4 and 8325-4ΔΔ*fnbAB* co-infecting with PA14, at an inoculum of $2x10^6$ CFU (**D**). Statistical significance of the trendline of all strains co-infections was determined with a linear regression test. Yellow dots represent NRS149 co-infections; orange dots represent NRS155 co-infections; red dots represent Newbould co-infections; purple dots represent NewbouldΔ*sigB* co-infections; pink dots represent NewbouldΔ*hemB* co-infections; green dots represent SH1000 co-infections; teal dots represent 8325-4 co-infections; blue dots represent 8325-4 ΔΔ*fnbAB.*

Table S1. ***P aeruginosa*** **clinical strains characteristics**

						Impact on *S. aureus in vivo*		
						Previous study by Fugère *et al.*, 2014	Current study by Millette *et al.*, 2019	
**Strain ID**	**Isolation date**	**Patient ID**	**Visit**	**Mixed infection origin**	**HQNO production (µM)**	**Biofilm induction (OD_{560nm})**	**Growth kinetics**	**Petri dish co-culture**
PAC5A	2007-08-25	4	1	Yes, with CF5A-L	0,57	1,00		
PAC5B	2007-08-25	4	1	Yes, with CF5A-L	0,71	0,03		
PAC6A	2008-02-13	5	1	Yes, with CF6B-L	8,99	1,50		
PAC6B	2008-02-13	5	1	Yes, with CF6B-S	11,44	2,28	Antagonistic	Slow growing colonies
PAC18A	2008-04-08	15	1	Yes, with CF18A-L	0,03	0,04		
PAC18B	2008-04-08	15	1	Yes, with CF18A-S	0,06	0,33		
PAC22A	2008-04-15	17	1	Yes, with CF22A-L	2,88	1,74	Not antagonistic	Slow growing colonies
PAC33A	2008-06-10	15	2	Yes, with CF33A-L	0,04	0,18		
PAC33B	2008-06-10	15	2	Yes, with CF33A-L	0,04	0,43		
PAC39A	2008-07-01	5	2	Yes, with CF39A-S	11,82	2,12	Antagonistic	Slow growing colonies
PAC44A	2008-09-09	18	2	No	1,21	0,95		
PAC44B	2008-09-09	18	2	No	0,18	1,80		
PAC44C	2008-09-09	18	2	No	0,44	0,28		
PAC44D	2008-09-09	18	2	No	0,34	0,20		
PAC46	2008-09-10	4	2	No	0,42	0,27		
PAC54A	2008-09-23	1	3	Yes, with CF54A-L	0,09	0,19	Not antagonistic	Regular-sized colonies
PAC60	2008-10-28	28	1	No	2,99	2,40		
PAC61	2008-10-28	4	3	Yes, with CF61B-L	0,15	0,11		

PAC74	2009-01-06	5	4	No	12,57	2,53		
PAC76	2009-01-13	31	1	No	0,73	0,77		
PAC78A	2009-01-13	15	3	Yes, with CF78A-L	1,07	0,46		
PAC78B	2009-01-13	15	3	Yes, with CF78A-L	0,13	0,10		
PAC84	2009-02-10	17	3	Yes, with CF84A-L	1,65	2,01		
PAC93A	2009-06-16	5	5	No	5,45	0,71		
PAC93B	2009-06-16	5	5	No	3,52	1,04		
PAC93C	2009-06-16	5	5	No	5,23	0,79		
PAC94A	2009-06-16	17	4	No	1,20	1,03		
PAC95A	2009-05-26	15	4	Yes	2,88	0,20		
PAC108	2009-11-17	18	3	No	0,52	1,94		
PAC112A	2009-12-15	31	2	Yes, with CF112A-L	7,23	1,93	Antagonistic	Slow growing colonies
